# Spatiotemporal patterns and ecological consequences of a fragmented landscape created by damming

**DOI:** 10.7717/peerj.11416

**Published:** 2021-05-21

**Authors:** Guang Hu, Maxwell Wilson, Bing-Bing Zhou, Chenwei Shang, Mingjian Yu, Jianguo Wu

**Affiliations:** 1 School of Civil Engineering and Architecture, Zhejiang Sci-Tech University, Hangzhou, China; 2 School of Life Sciences, Arizona State University, Tempe, Arizona, United States; 3 School of Sustainability, Arizona State University, Tempe, Arizona, United States; 4 School of Natural Resources, Faculty of Geographical Science, Beijing Normal University, Beijing, China; 5 Center for Human-Environment System Sustainability (CHESS), Beijing Normal University, Beijing, China; 6 College of Life Sciences, Zhejiang University, Hangzhou, China

**Keywords:** Damming, Landscape fragmentation, Habitat loss and fragmentation, Landscape dynamics, Biodiversity, Thousand Island Lake, Socioeconomic changes, Ecological impacts

## Abstract

**Background:**

Damming disrupts rivers and destroys neighboring terrestrial ecosystems through inundation, resulting in profound and long-lasting impacts on biodiversity and ecosystem processes far beyond the river system itself. Archipelagos formed by damming are often considered ideal systems for studying habitat fragmentation.

**Methods:**

Here we quantified the island attributes and landscape dynamics of the Thousand Island Lake (TIL) in China, which is one of the several long-term biodiversity/fragmentation research sites around the world. We also synthesized the major findings of relevant studies conducted in the region to further ecological understanding of damming and landscape fragmentation.

**Results:**

Our results show that the vegetations on islands and the neighboring mainland were both recovering between 1985 and 2005 due to reforestation and natural succession, but the regeneration was partly interrupted after 2005 because of increasing human influences. While major changes in landscape composition occurred primarily in the lakefront areas and near-lakeshore islands, landscape patterns became structurally more complex and fragmented on both islands and mainland. About 80 studies from the TIL region show that the genetic, taxonomic, functional, and phylogenetic diversity on these islands were mainly influenced by island area at the patch scale, but fragmentation per se also affected species composition and related ecological processes at patch and landscape scales. In general, islands had lower species diversity but a steeper species-area relationship than the surrounding mainland. Fragmentation and edge effects substantially hindered ecological succession towards more densely vegetated forests on the islands. Environmental heterogeneity and filtering had a major impact on island biotic communities. We hypothesize that there are multiple mechanisms operating at different spatial scales that link landscape fragmentation and ecological dynamics in the TIL region, which beg for future studies. By focusing on an extensive spatiotemporal analysis of the island-mainland system and a synthesis of existing studies in the region, this study provides an important foundation and several promising directions for future studies.

## Introduction

Dams and reservoirs have played an important role in human’s history for water resource management, such as hydropower generation, flood control, and water supply (Baxter, 1977). By 2018, a total of 59,071 large dams (height > 15 m) were built worldwide, about 40% of which (23,841) were located in China (ICOLD, 2018). Damming disrupts the river flow and physically modifies the landscape, resulting in overland inundation that turns previously terrestrial ecosystems into lakes or reservoirs (Schmutz & Moog, 2018). In regions with complex topography, dam construction often transforms former hilltops or mountaintops into land-bridge islands surrounded by water (Kingsford, 2000), forming an ideal study system for the impacts of habitat fragmentation and landscape fragmentation on biodiversity and ecosystem properties (Gibson et al., 2013; Hu et al., 2011; Jones et al., 2016; Liu et al., 2019a; Wu, Huang & Han, 2003a; Wu et al., 2004; Wu et al., 2003b). While dam construction may be a short-term local event, its destructive impacts on natural habitat, biodiversity, ecosystem processes, and landscape patterns are long-lasting in time and far-reaching in space (Ligon, Dietrich & Trush, 1995; Rodrigues & Silva, 2012; Terborgh et al., 2001; Wu, Huang & Han, 2003a; Wu et al., 2004; Wu et al., 2003b; Zhao et al., 2012). Because of their clear physical boundaries, homogeneous matrix, and strong isolation (Wu et al., 2003b), a number of worldwide studies have examined the ecological consequences of damming-created islands across different taxon (Table 1) in recent decades. On such land-bridge islands, ecologists observed “ecological meltdown” in Venezuela (Terborgh et al., 2001; Terborgh, Lopez & Tello, 1997), “ecological relaxation” in Wales (Greenwood et al., 1999), “extinction debt” in reservoir islands worldwide (Jones et al., 2016), and a number of other biodiversity and ecosystem responses to habitat fragmentation (Chen et al., 2019; Hernandez & Sandquist, 2019; Liu et al., 2019b; Wilson et al., 2020).

**Table 1:** Main dam-caused reservoirs with studies of landscape fragmentation across world.

Reservoir	Country	Completion year	Area (km^2^)	Biome	Studied taxon						
					Plant	Bird	Insect	Reptile	Mammal	Amphibian	Fish
Lake Gatún	Panama	1912	5400	Tropical forest	√				√		√
Thousand Island Lake	China	1959	580	Subtropical forest	√	√	√	√	√	√	√
Cabra Corral	Argentina	1972	133	Subtropical forest			√				√
Tucurui	Brazil	1984	2918	Tropical forest	√	√	√		√	√	√
Lake Kenyir	Malaysia	1985	370	Tropical forest	√	√	√				√
Chiew Larn	Thailand	1986	165	Tropical forest		√			√		
Lago Guri	Venezuela	1986	4300	Tropical forest	√	√	√				
Balbina	Brazil	1989	4437	Tropical forest	√	√	√	√	√		√
Petit Saut	French Guiana	1994	365	Tropical forest	√		√	√	√		√

While these island studies have provided much insight into how habitat fragmentation affects biodiversity and ecological processes, little is known about the ecological effects of within-island spatial heterogeneity and surrounding regional landscapes (Schiemer et al., 2020; Wilson et al., 2020). Numerous empirical studies on landscape fragmentation and patch dynamics in modern landscape ecology (Turner & Gardner, 2015; Wu & Loucks, 1995) suggest that such effects are more than likely to be important one way or another. Indeed, some recent reservoir island studies suggest that island pattern and dynamics, as well as surrounding mainland landscapes, may play an important role in regulating the local ecological patterns and processes on individual islands (Ding et al., 2004; Hu et al., 2012; Schiemer et al., 2020; Wilson et al., 2020). Distances among islands and between islands and the mainland may also influence population processes and thus species composition on islands (Hu et al., 2019; Yu et al., 2012), which implies that a landscape ecological approach explicitly considering both within- and beyond-island spatial heterogeneity would be necessary (Wilson et al., 2020). Here we note that the two terms–habitat fragmentation and landscape fragmentation–are related but distinct concepts: the former refers to breaking up of habitat, while the latter to breaking up of landscape that may include both habitat and non-habitat elements.

To further our understanding of the ecological impacts of damming and habitat/landscape fragmentation, here we conducted the first historical landscape analysis of the Thousand Island Lake (TIL) in southeastern China, an artificial lake with more than a thousand islands and one of the several long-term biodiversity/fragmentation research sites around the world (Wilson et al., 2016). Based on the existing literature and additional analysis using remote sensing data and survey records, we also synthesized the major ecological findings of existing studies conducted in the TIL region. In doing so we answer the following questions: (1) what are the long-term landscape and ecological consequences of damming in this fragmented landscape; and, (2) how has socioeconomic development affected the landscape dynamics, ecosystem processes, and community dynamics after damming. In addition, we proposed a landscape ecology-based framework to link regional landscape dynamics with ecological processes for better understanding and managing the ecology and sustainability of the island-mainland complex as a coupled human-environment system.

## Materials & methods

### Study area

**Figure 1:**
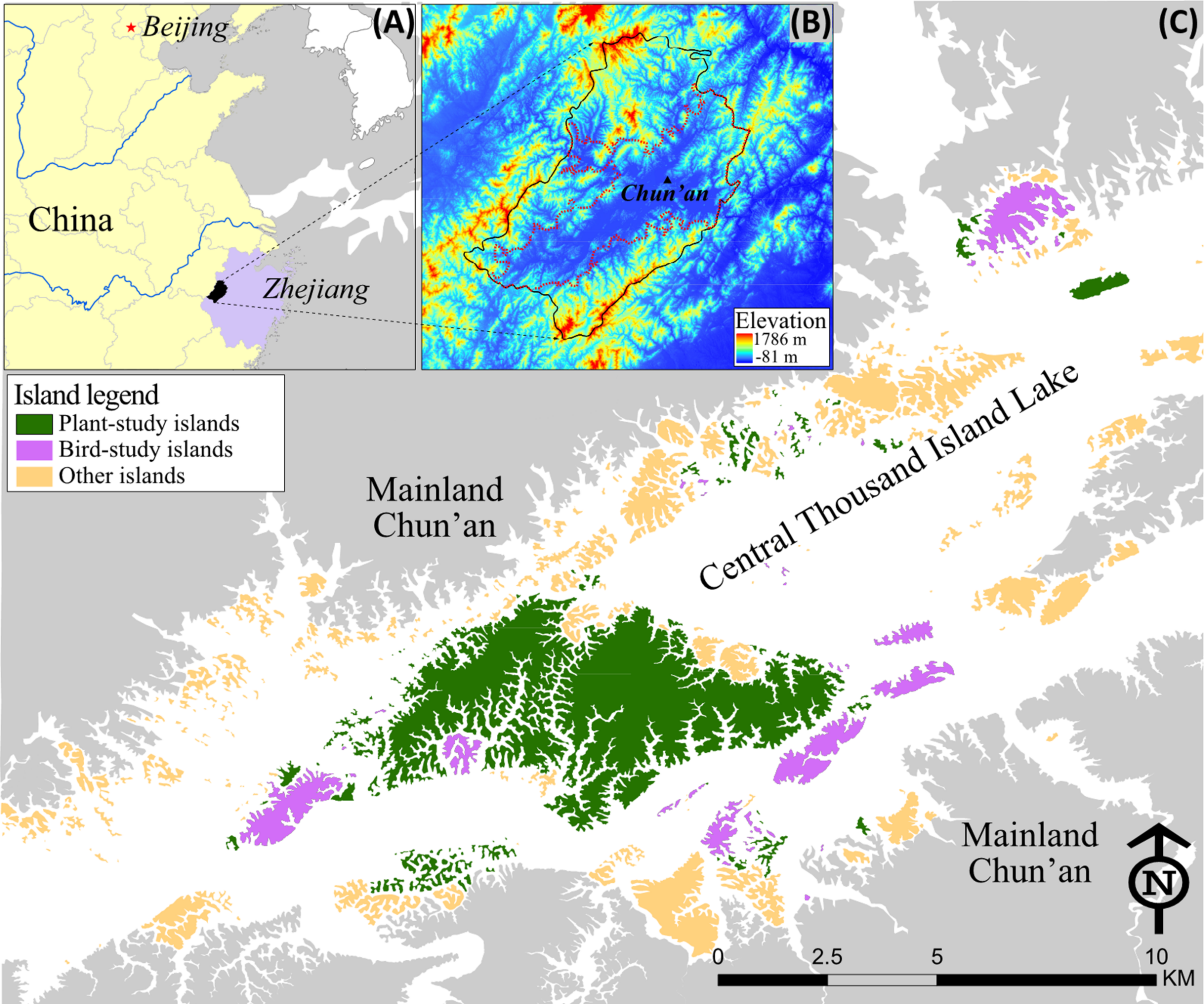
Map of the Thousand Island Lake (TIL) region. (A) Location of TIL in Chun’an County, Zhejiang Province of China; (B) Elevation map of Chun’an (black boundary) and the TIL national forest park (red boundary); (C) TIL islands that have been studied, with all the bird-study islands overlapping with plant-study islands.

The Thousand Island Lake (TIL) is located in Chun’an County of Zhejiang Province, China (29°11′‒30°02′N, 118°34′‒119°15′E; Fig. 1A), which is best known for its spectacular scenic landscapes, having attracted millions of tourists from China and worldwide since the 1990s. TIL was created when the Chinese government built the Xin’an River Hydroelectric station by damming during 1956-1959. The lake runs 150 km from southwest to northeast, with a total area of 581 km^2^, an average depth of 30.4 m, and a water storage of 17.8 billion m^3^ (Fig. 1B). As one of the reservoirs with a massive number of islands in the world, TIL has 1,078 islands (>0.25 ha) with a total area of 40.9 km^2^ at the designed highest water level of 108 m above the sea level. Submerged under water were two ancient cities of more than 1300 years (He Cheng built in 208 AD during Han Dynasty and Shi Cheng built in 621 AD during Tang Dynasty), along with 49 towns, 1,377 villages, thousands of residential homes, and over 200 km^2^ of farmland. About 290,000 residents were relocated by the government due to the dam’s construction (Editorial Committee of Chun’an County Annals, 1990).

Under the prevailing subtropical monsoon climate, the mean annual temperature in the TIL region varies from 17 °C to 18 °C, with a frost-free season of 241~296 days. The mean annual precipitation varies from 1148 to 2015 mm. The typology of Chun’an is basin-shaped, with mountains on all sides. Prior to the lake formation in the late 1950s, forests in this region including the current lake surface, islands, and mainland adjoining the reservoir, were completely clear-cut. The contemporary forests on the islands are of similar age (approximately 60 years old), roughly corresponding to the end of the Great Leap Forward (1958–1960) and the implementation of immigration policies for the local people. Native pines from local areas quickly regenerated themselves on these islands as pioneer species. Most of the islands (previously mountaintops) were disturbed by human activities in the past, but the TIL region has been protected since 1986 as the largest national forest park, Thousand Island Lake National Forest Park (Fig.1B) in China (Fang & Yu, 1996). From 1963 to 2013, the forest coverage of the TIL region increased from 38.6% to 87.0% (Xu et al., 2016). The TIL region is rich in biodiversity, including 666 species of vascular plants, 177 bird species, 18 reptile species, 9 amphibian species, 20 mammal species, and 428 arthropod species (Xu et al., 2015).

TIL is the main drinking water resource for the downstream cities. The environment and wildlife have been protected by national and local laws (National Development & Reform Commission of China, 2013; National People’s Congress of China, 1989; State Forestry Administration of China, 2011; The People’s Government of Zhejiang Province, 2019). Although tourism is the main source of income of this region, only a dozen islands have been developed for tourism since 1981 (Editorial Committee of Development of Xin’an River, 2009). In 2016, about 1.26 million tourists visited TIL and contributed 12 billion RMB to the local economy (Editorial Committee of Chun’an Local History, 2017). The policies and actions for tourism development and environment conservation have created a landscape mosaic of islands of different purposes near the lakeshore of TIL. During the recent decades, development pressures seem to increase locally and regionally, and landscape transformations of surrounding mainland areas have become faster and more intense than ever before.

### Remote sensing data for quantifying key features of the central TIL islands

Three key island features‒island area, Euclidean nearest neighbor distance (ENN), and distance to mainland‒were quantified for the central TIL’s archipelago (Fig. 1C) where most existing ecological studies were carried out (Ding et al., 2015; Hu et al., 2011; Hu et al., 2019; Hu et al., 2012; Wang et al., 2010a; Yu et al., 2012). To ensure accuracy, a SPOT-6 satellite imagery with a spatial resolution of 1.5 m (taken on October 13, 2013, pan-sharpened and orthorectified in natural color) was acquired for digitalizing islands and mainland borders based on visual interpretation using ArcGIS. Island boundaries were defined as the vegetated edge of each island, as island area is dependent on water level (ranging from 80 to 108 m a.s.l.; 105 m was mostly treated as the standard water level measuring the island area). The resulting map was then rasterized for calculation of island area and ENN in FRAGSTATS 4.2 (McGarigal, Cushman & Ene, 2012), while distance to mainland was calculated using the Near tool in ArcGIS. Besides ENN and distance to mainland, distance to the nearest larger island and island shape were also used as the proxies linking to the fragmentation per se at patch scale. Meanwhile, the patch number, patch density, and the relative habitat amount were used to measure the fragmentation degree at the landscape scale in previous studies in the TIL region (Hu et al., 2012; Jin et al., 2020; Wilson et al., 2020).

### Land use/cover products for analyzing landscape dynamics

Classified land use/cover (LUC) data of Chun’an for the years 1985, 1990, 1995, 2000, 2005, 2010, and 2015 (see Appendix 1 for the raw data) were used for this study. These data are standard products from the Resources and Environment Sciences Data Center, Chinese Academy of Sciences (http://www.resdc.cn/), and have been widely used in studying China’s LUC changes (e.g., Deng et al., 2015; Liu et al., 2002; Liu et al., 2005). The LUC data were produced using Landsat imagery, with a resolution of 30 m and the overall accuracy above 90% (Liu et al., 2014). The LUC types were originally classified into six first-level classes: cropland, woodland, grassland, water body, built-up land, and unused land, with 25 second-level classes (see Liu et al. (2005) for details).

**Figure 2:**
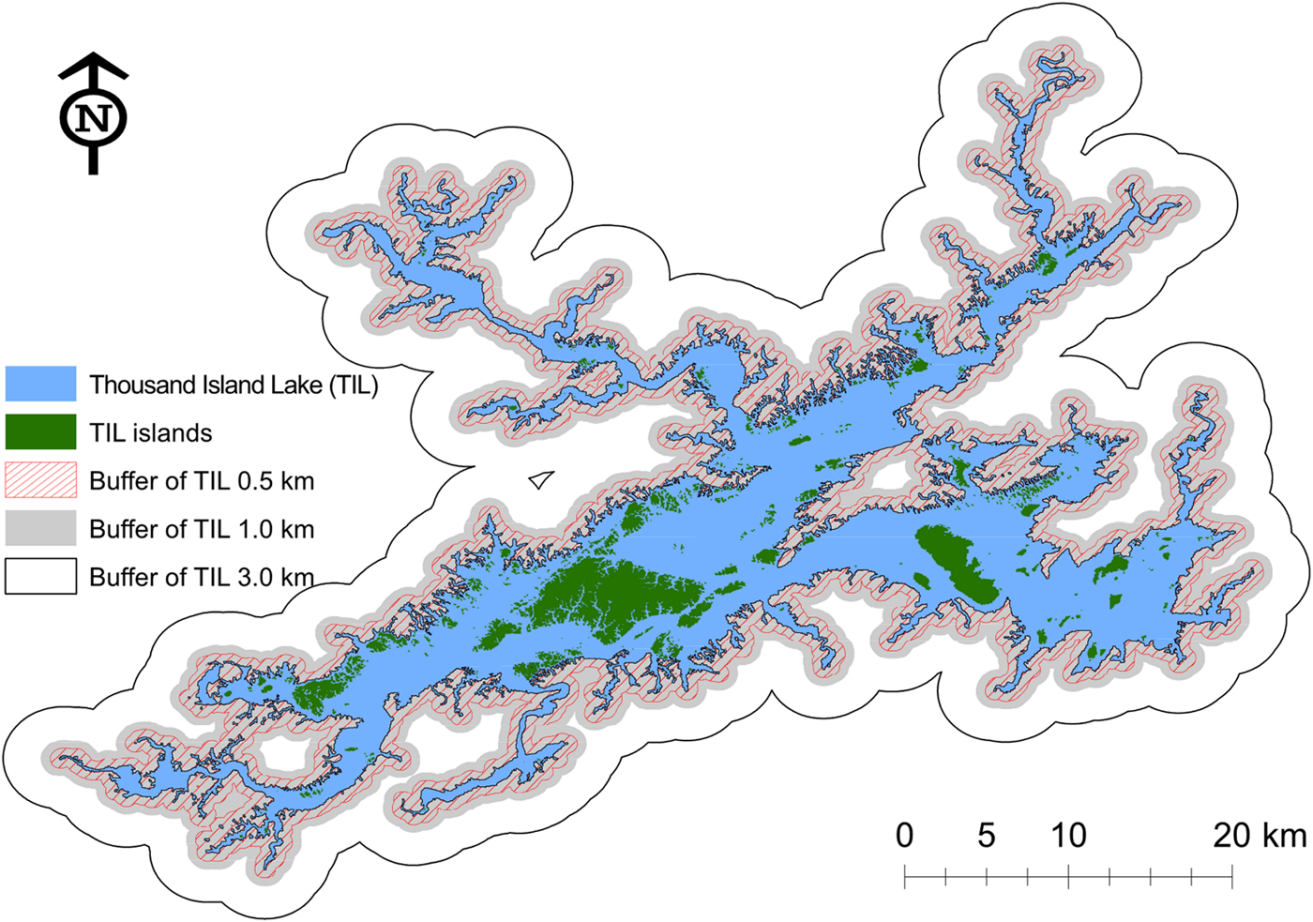
Illustration of three mainland buffers with widths of 0.5, 1.0, and 3.0 km, used for the landscape pattern analysis. Larger buffers contain smaller ones, together forming a spatially nested hierarchy.

In this study, the LUC classes were reclassified into seven types in ArcGIS 10.2 following the reclassification scheme in Zhou & Lv (2020): agricultural lands, industrial and transportation lands, rural settlement, urban built-up lands, sparsely-vegetated natural lands (i.e., woods, other woodland, moderate grass, sparse grass, sandy land, bare soil, bare rock, and other unused land), densely-vegetated natural lands (i.e., forest, shrub, and dense grass), and water bodies. To compare the LUC dynamics between TIL islands and the surrounding mainland landscapes, three buffers of 0.5, 1.0, and 3.0 km were created in ArcGIS starting from the TIL-mainland border (Fig. 2). The buffers were spatially nested–smaller ones were contained by larger ones. Subsequently, the landscape dynamics were quantified both compositionally and configurationally at the class level using R package ‘SDMTools’ (Brown, 2014). We adopted several landscape metrics to capture changes in landscape composition and configuration, focusing on amount and adjacency (Riitters, 2019). These pattern metrics include class area and proportion, mean and standard deviation of patch area, mean and standard deviation of fractal dimension index, patch density, and aggregation index (McGarigal, Cushman & Ene, 2012).

### Assessing ecological impacts of habitat fragmentation from TIL studies

To advance our understanding of how habitat fragmentation affects biodiversity and ecosystem processes, we collected all relevant published studies conducted in the TIL region using Web of Science and Google Scholar (see Appendix 2 for the full list of publications). Thematic mapping by VOSviewer (van Eck & Waltman, 2010) was conducted to illustrate the main research themes covered by the existing TIL studies. Furthermore, full-texts of the publications were reviewed qualitatively in terms of the taxa investigated (e.g., plants, birds, amphibians, reptiles, insects, spiders, and mammals) and the different kinds of diversity examined (e.g., taxonomic, functional, phylogenetic, and genetic diversity). Other ecological consequences such as the impacts on succession, species interaction, population demography, and soil condition were also reviewed.

In addition, we computed the Normalized Difference Vegetation Index (NDVI) between 1985 and 2011 based on Google Earth Engine (GEE) and associated atmospherically corrected surface reflectance images of Collection 1 Tier 1 from the Landsat ETM 5 sensor. NDVI is commonly used to identify the vegetated land and evaluate the habitat’s characteristics, for example, net primary productivity, forest health, or phenology. In this study, we selected NDVI as a comprehensive proxy for assessing habitat quality (Pettorelli et al., 2005), and compared across the time series to assess the spatiotemporal pattern of the vegetated land in this region. For NDVI comparability, images from other Landsat sensors were not included. In total, there were about 1140 images (roughly 570 Gigabytes) processed, with the Quality Assessment 16-Bit band in each image used for removing clouds or cloud shadows. The output NDVI of each pixel was the maximum value composite on an annual basis (see Appendix 3 for the GEE code). The 1985 and 1986 NDVI maps were excluded from analysis, however, because of severe impacts of clouds, thus resulting in a 25-year time-series (i.e., 1987–2011) of annual NDVI maps. To compare the NDVI spatiotemporal patterns of TIL islands and the mainland, the three mainland buffers of 0.5, 1.0, and 3.0 km were also analyzed in the LUC analysis. The Wilcoxon rank test was used to compare the median NDVIs among different zones (islands and three mainland buffers), to explore the spatial pattern of habitat quality. The Mann-Kendall Test and the Pettitt’s Test were performed on the four time-series to detect any temporal trend or change point. The coefficient of variation (CV) was used to evaluate the temporal variations. Linear regression was also conducted to test the relationship between the NDVI of the zones. All the above-mentioned statistical tests were implemented in R (R Core team, 2019).

### Socioeconomic data for the TIL region

Socioeconomic data for the TIL region were collected mainly from the Chun’an yearbooks in CNKI Database (https://oversea.cnki.net/knavi/YearbookDetail?pcode=CYFD&pykm=YDISH). Additional qualitative information from 1955 to 2017 was obtained from the Hangzhou yearbooks (https://oversea.cnki.net/knavi/YearbookDetail?pcode=CYFD&pykm=YANNF) and reports, and major socioeconomic events were identified as potential key drivers for landscape dynamics in the region. The collected quantitative data include population, Gross Domestic Products (GDP), percentage of the primary industry’s GDP, and GDP per capita, ranging from 1978 to 2017, as well as tourism income and tourist amount from 1997 to 2017. The Mann–Kendall Trend Test and the Pettitt’s Test for Change-Point Detection were conducted on time-series to determine how the population, the economy, and the TIL-based tourism have been changing in the past few decades.

## Results

### Landscape dynamics of the TIL-mainland system

**Figure 3:**
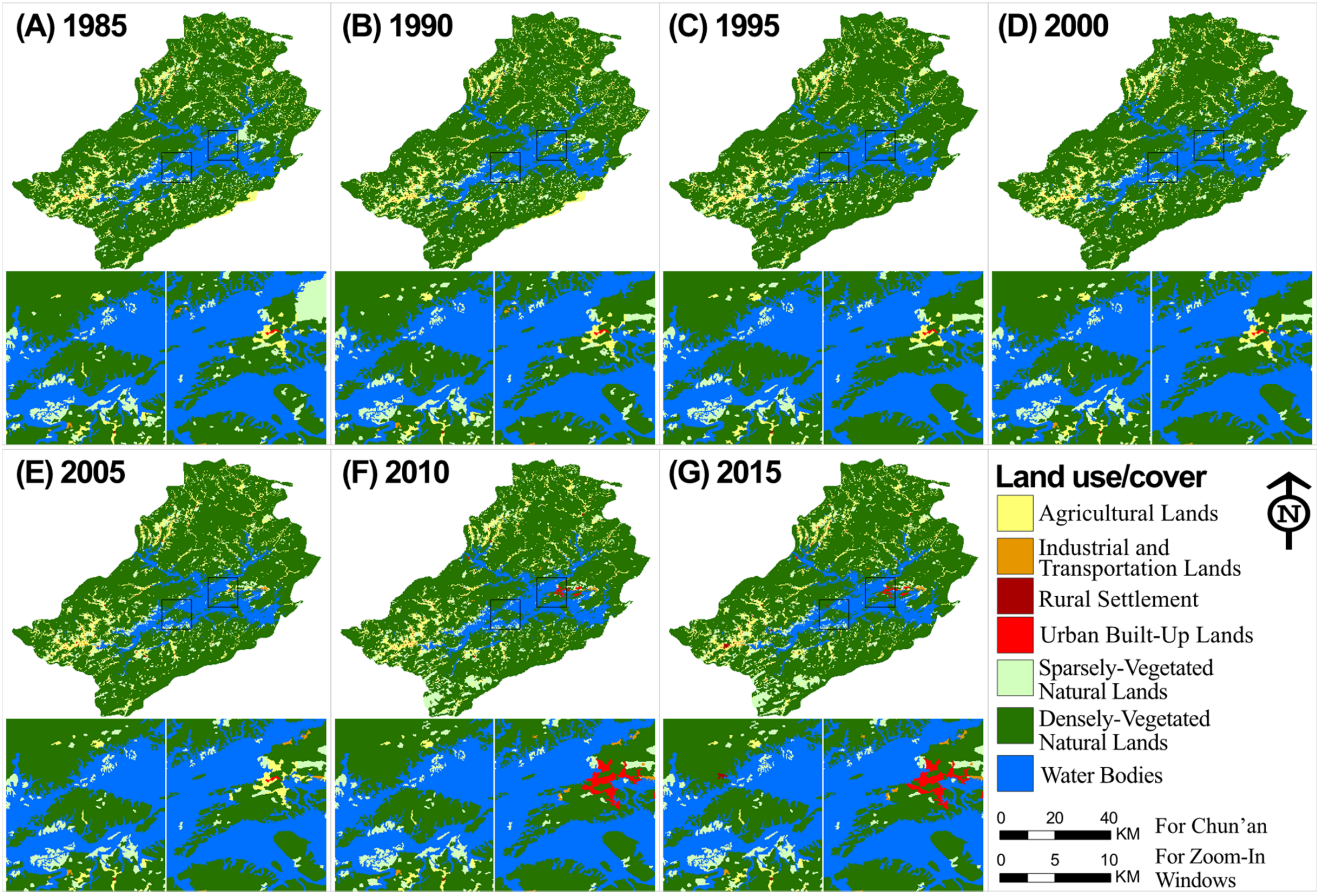
Land use and land cover change in the TIL region from 1985 to 2015. The zoom-in views show the Jieshou Island (the biggest island of TIL) and Downtown Chun’an where the county government is located. (A)-(G) Land use and land cover of TIL region at 1985,1990, 1995, 2000, 2005, 2010, and 2015.

Landscape dynamics of the TIL-mainland system show two distinct stages (Fig. 3): the vegetation on the landscape was recovering (e.g., increases in number of green pixels on the RS imagery) from 1985 to 2005, but replaced by urban expansion (e.g., increases in number of red pixels) after 2005. There was noticeable reforestation in mainland Chun’an during 1985–2005 and on TIL islands during 1995-2005, in accordance with China’s nationwide reforestation movement in the 1990s. The reforestation occurred mainly on sparsely-vegetated natural lands. Since 2005, downtown Chun’an has been increasingly urbanized; and, especially after 2010, the trend of landscape urbanization expanded further to western Chun’an (Fig. 3). The observable qualitative landscape changes suggest a potential socioecological regime shift following fast infrastructure construction at around 2005 (detail showed in following context).

**Figure 4:**
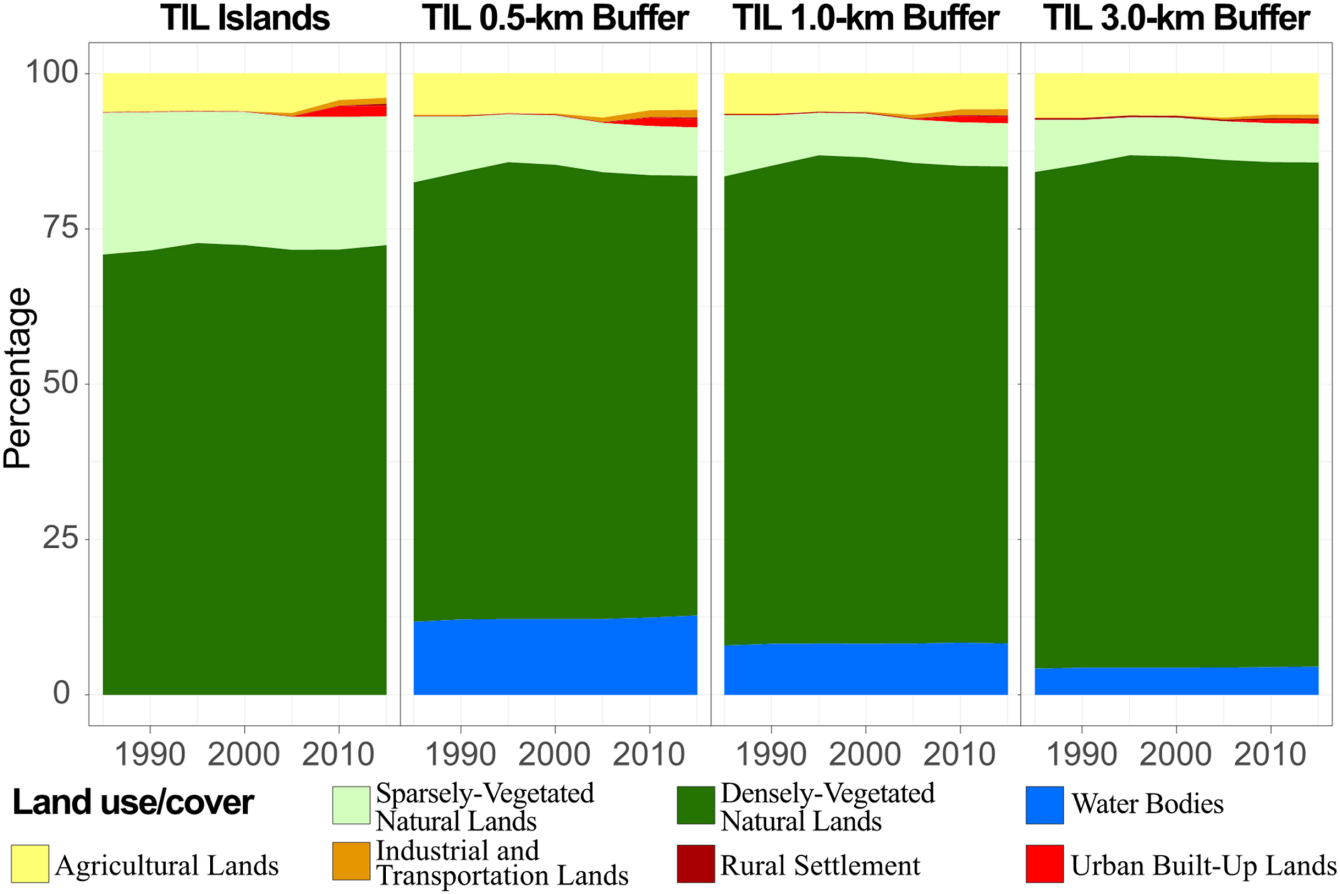
Changes in landscape composition of TIL islands during 1985–2015, with three mainland buffers of 0.5, 1.0, and 3.0 km in width. All went through the land use transition of reforestation-stabilization-urbanization, with TIL islands having more sparsely-vegetated and less densely-vegetated natural lands.

Beyond our expectation, the landscape composition-the proportion of the landscape for each land cover class-of the well protected islands performed a similar pattern and trend with those of the disturbed mainland (Fig. 4). While islands accounted for about 7.2% of the lake area, around 73% of the TIL national forest park (Fig. 1B) was covered by water surface. The islands and mainland were both mainly covered by densely-vegetated natural lands (~70%) and sparsely-vegetated natural lands (15%–25%). When the width of mainland buffers increased from 0.5 to 1.0 and 3.0 km, the percentage of densely-vegetated natural lands increased from 71% to 81%. After 1995, the relative abundance of densely-vegetated natural lands was quite stable on the TIL islands and mainland. Also, changes in the landscape composition of TIL islands and the mainland were in line with the observation that landscape dynamics in this region was dominated by reforestation before 1995 and by urbanization after 2005 (Fig. 4). This regenerated vegetation supported a large species pool (Xu et al., 2015) which could maintain the regional biodiversity. Additionally, the TIL islands and lakeshores (0.5 km buffer) had a higher proportion of human-dominated land uses such as urban built-up lands and industrial and transportation lands especially after 2005. This is significant, as natural areas on the lakeshore may serve as immigrant pools for island biological communities (Yu et al., 2012).

**Figure 5:**
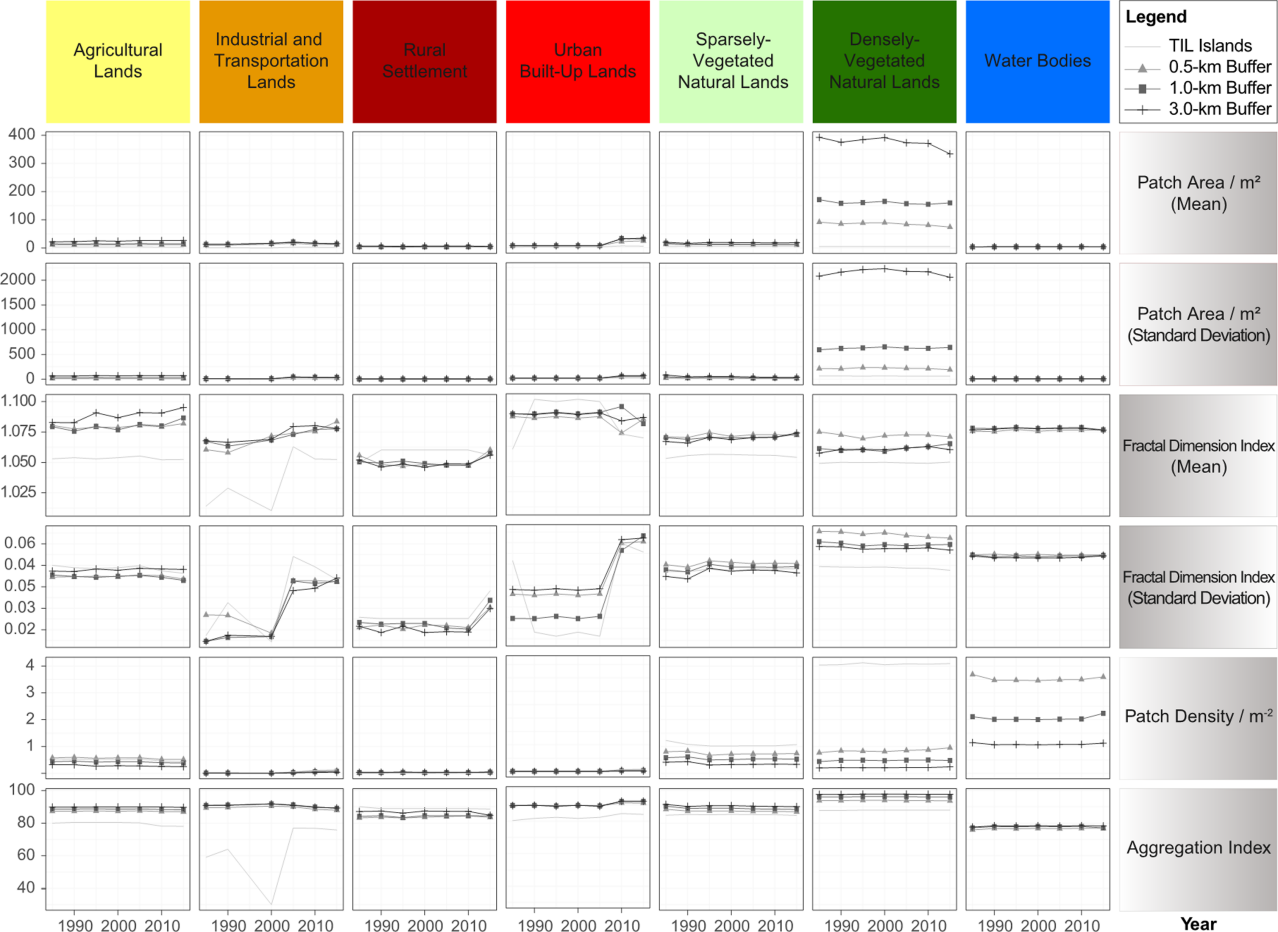
Changes in landscape configurational features of TIL islands and the surrounding mainland during 1985–2015, with three mainland buffers of 0.5, 1.0, and 3.0 km in width. Natural lands of TIL islands were the most fragmented and least disturbed.

However, landscape configurational features, such as mean patch area, patch density, patch shape, and patch aggregation, showed much smaller variations across time and between the TIL islands and the mainland (Fig. 5). In particular, mean patch area had little temporal variation for all LUC types, except urban land and densely-vegetated natural lands (Fig. 5). Most noticeably, all human-dominated LUC types (transportation, rural and urban lands), except agricultural lands, had an increasing trend in patch shape complexity (indicated by fractal dimension measures). The patch shape complexity of natural lands did not change appreciably, with TIL islands having lower values of fractal dimension index than the mainland buffers, which indicates stronger human disturbances on the mainland than TIL islands. Patch density and aggregation index did not change much over time, but TIL islands generally had lower values of aggregation index and higher values of patch density than the mainland (Fig. 5). Lower means of these indices indicated that TIL islands were internally more fragmented, but lower standard deviations revealed the LUC dynamics on islands were more stable and less disturbed (especially for vegetated natural lands) than the surrounding mainland.

### Ecological impacts of landscape fragmentation on TIL islands

**Figure 6:**
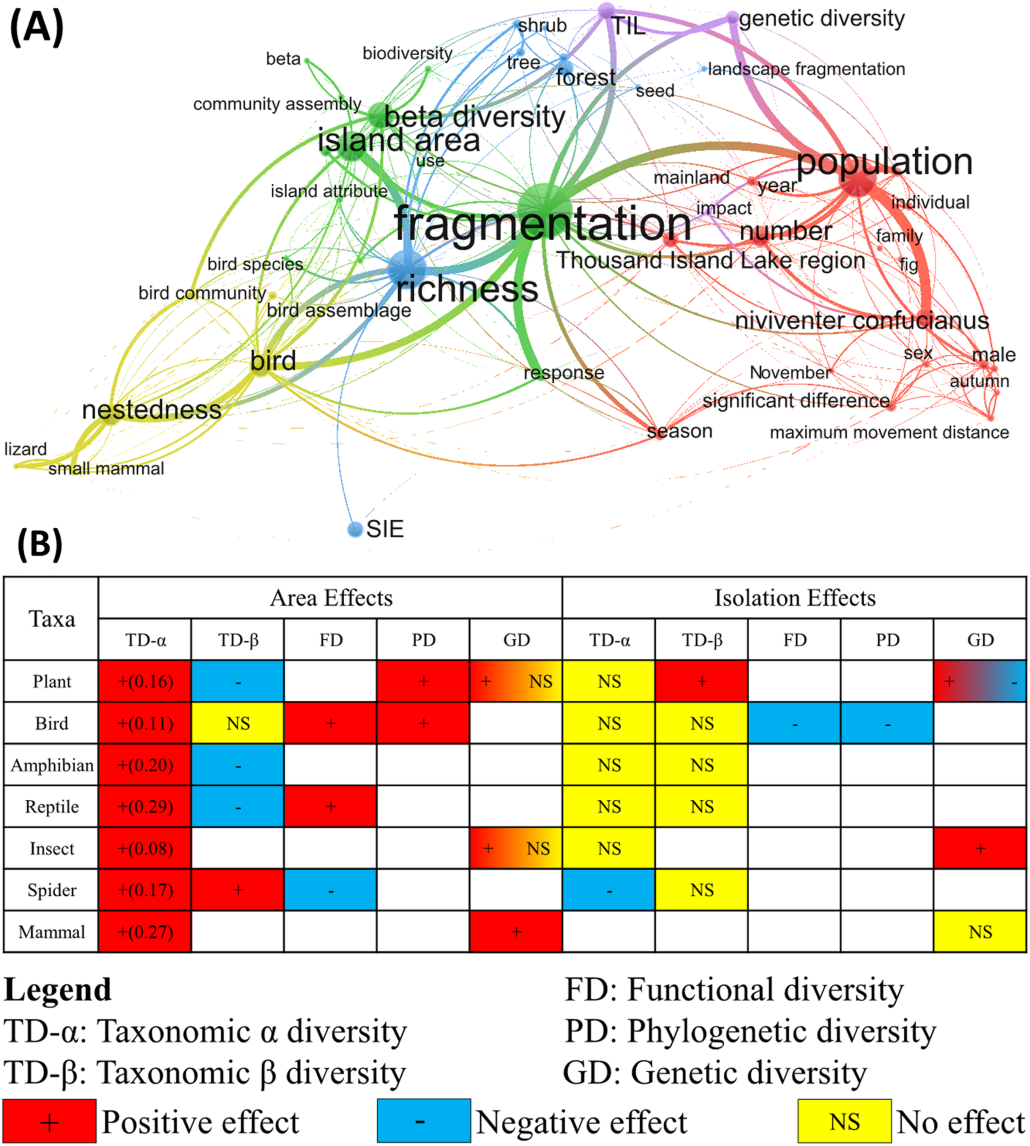
Existing studies and findings on ecological impacts of habitat fragmentation in the TIL region. (A) clusters of the topics extracted from the titles and abstracts of existing studies conducted in the Thousand Island Lake (TIL) region (see Appendix 2 for the full list of publications). (B) Fragmentation effects (area effect and isolation effect) on biodiversity across taxa and organizational levels. The values in parentheses are the slopes of log-log transformed species-area curves.

Since 2004, a series of ecological studies on landscape fragmentation have been conducted in the TIL region. Our thematic mapping (Fig. 6A) revealed that these studies focused on two main topics: biodiversity and population responses to landscape fragmentation. For the first topic, the main research question was how island attributes affected biodiversity of different taxonomic groups at the plot and island scales (Hu et al., 2011; Ren et al., 2009; Si et al., 2014; Wang et al., 2015). Studies on the second topic involved mostly the population structure and behavior of a common rat (*Niviventer confucianus*) (Sun et al., 2009; Ye et al., 2013; Zhang et al., 2013; Zhang et al., 2016), as well as the genetic differentiation of certain plant species across islands (Liu et al., 2013; Luo et al., 2014; Ye et al., 2016; Yuan et al., 2015; Yuan et al., 2012).

These studies have shown a positive relationship between island area (equal to habitat area) and genetic, taxonomic, functional, and phylogenetic diversity across different taxa (Fig. 6B), but higher plant β diversity was found among small islands than large islands (Liu et al., 2018). Patch-scale species diversity was lower and species-area relations were steeper on islands than in the surrounding mainland (Wang, Chen & Ding, 2011; Wang et al., 2010a). Although isolation effects on biodiversity and ecological processes on the TIL islands were generally minor, some significant relationships between isolation (or fragmentation per se) and species composition (Fig. 6) were detected (Hu et al., 2019; Yu et al., 2012). The species composition, in addition to species diversity, of island plant communities was also affected by habitat loss and fragmentation after damming. For instance, enhanced edge effects due to fragmentation caused the environmental degradation on small islands, and supported more early-successional plant species (Liu et al., 2019b). Species interactions, such as seed dispersal, predation and herbivory, within and between islands were also influenced by habitat loss and fragmentation. Density of bird-dispersed plants with smaller seed sizes was significantly positively related with island area (Liu et al., 2019c). Common species with higher defensive abilities and lower nutrient contents on the islands suffered less hebivory (Luo et al., 2017). The seed predation rate was higher on larger and less isolated islands (Li et al., 2013; Tong et al., 2017). In addition, lower soil fertility was measured on these fragmented islands than the surrounding mainland (Rong et al., 2018).

At the landscape scale, the species-area curve became steeper in the more fragmented landscapes, which was measured by habitat amount and patch number (Hu et al., 2012; Wilson et al., 2020). Studies also have also revealed that the effects of habitat fragmentation on biodiversity were scale-dependent, with hierarchical species-habitat linkages (Wilson et al., 2020) and cross-scale interactions affecting plant functional diversity (Jin et al., 2020).

**Figure 7:**
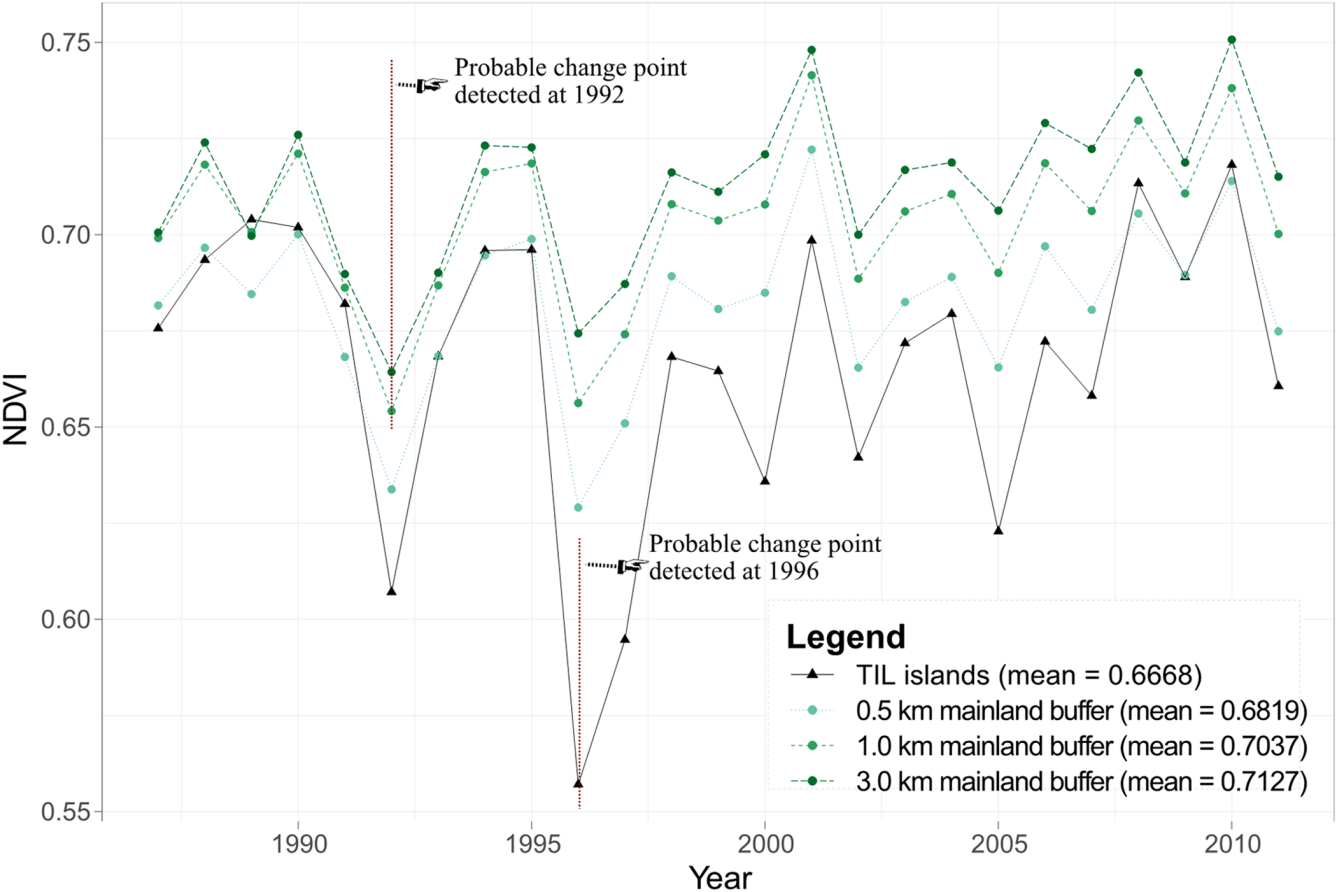
Changes in NDVI of islands and three mainland buffers with widths of 0.5, 1.0, and 3.0 km in the TIL region between 1985 and 2011.

Dam construction also influenced the distribution of NDVI, which is commonly used as a measure of habitat quality (Pettorelli et al., 2005), over the entire fragmented landscape. Wilcoxon rank test revealed that the NDVI time series of the TIL region could be divided into three groups (a, islands; b, 0.5 km mainland buffer; c, 1.0 and 3.0 km mainland buffers), with significant inter-group differences. NDVI tended to increase from the TIL islands onward to the mainland (Fig. 7). The strongest temporal fluctuations in NDVI were observed for the islands (CV = 5.83%). The results of the Mann-Kendall Test and the Pettitt’s Test showed an initial decreasing trend of islands’ NDVI, and turn to increase after 1996 (*S*_*island*_ = 52.00, *P*_*island*_ = 0.022), while such a reversal trend occurred earlier at 1992 on mainland (*S*_*1km*_ = 76.00, *P*_*1km*_ = 0.023; *S*_*3km*_ = 81.00, *P*_*3km*_ = 0.016) for the 1.0 and 3.0 km buffers (Fig. 7). Although no significant change point of the lakeshore’s NDVI (0.5 km buffer) was detected, we found a strong linear relation between island’s and lakeshore’s NDVI (*R*^*2*^= 0.779, *P* < 0.001).

### Major socioeconomic changes in the TIL region

From the inundation caused by dam construction in 1959 to the beginning of China’s “Reform and Opening-Up” in 1978, socioeconomic conditions shaped by central and local governments’ policies changed substantially in the TIL region, but so far have not been well-documented. A common narrative publicly available is that society and economy stagnated during the ten years of “Cultural Revolution” (1966–1976). In 1982, the government of Chun’an County established a state-owned enterprise to develop and manage the TIL region; in the same year, Guihua Island became the first island to be developed, and officially open for tourism (Editorial Committee of Development of Xin’an River, 2009). The TIL Administration was founded in 1985, and established a protected national forest park to include TIL and its surrounding mainland areas in 1986. The economic growth of the TIL region had been slow until 1992 when the TIL Economic Development Zone was created in response to Deng Xiaoping’s call for promoting a market-based economy in China. However, the TIL region’s development remained relatively slow until around 2005, when transportation infrastructure started to expand quickly. After that, hotels, resorts, and villas mushroomed along the lakefront, especially during 2008–2011.

**Figure 8:**
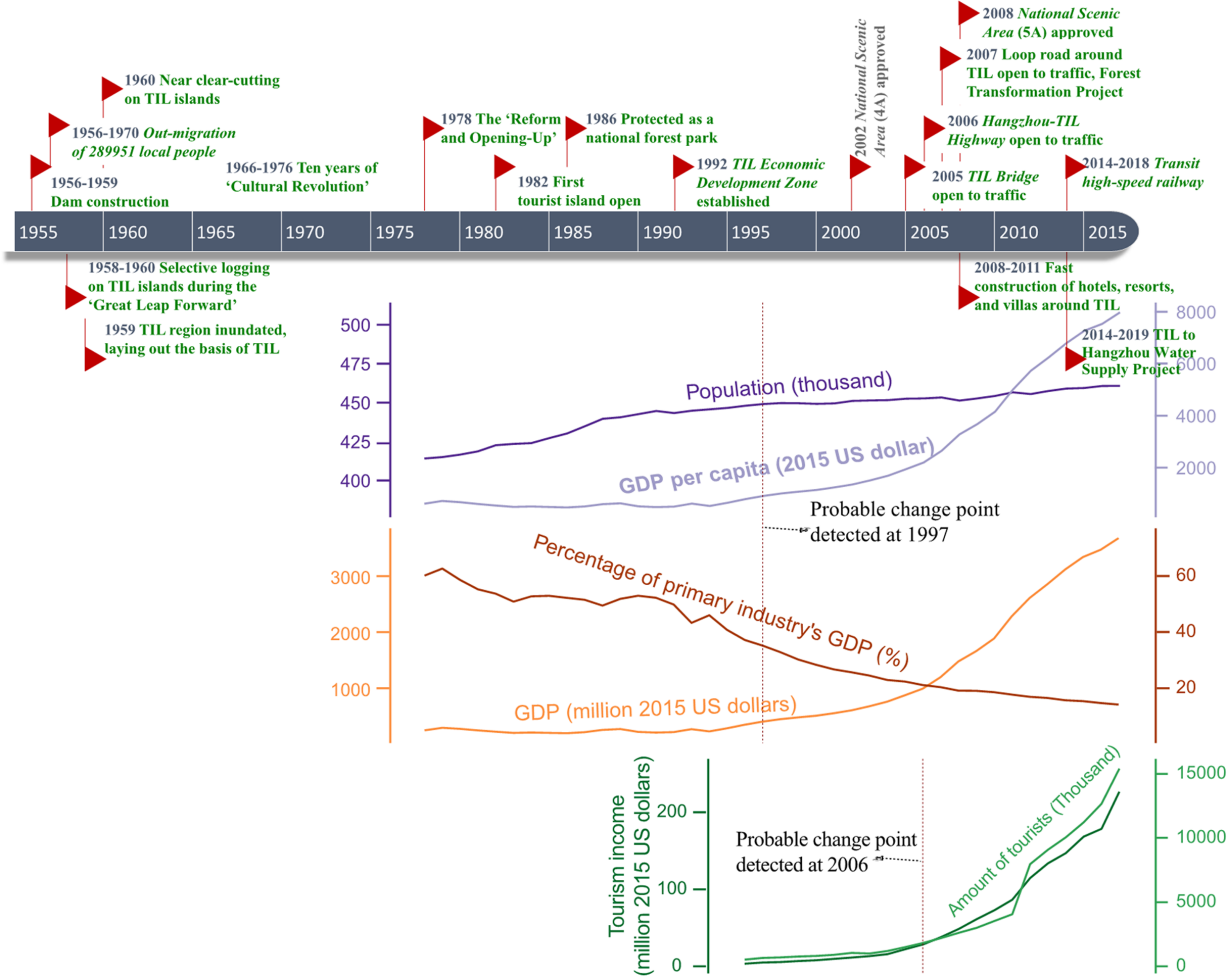
Schematic chronology of major national and local policies as well as socioeconomic events and conditions in the TIL region from 1955 to 2018.

According to yearbooks and socioeconomic statistics from governmental sources, population, economy, and tourism in Chun’an County all have showed an upward trend since 1978, influenced by a number of national and local policies (Fig. 8). A change point in population, GDP per capita, GDP, and percentage of primary industry’s GDP was statistically detected in 1997, when the Asian Financial Crisis broke out and China started to make long-term, large-scale, and nationwide investments on infrastructure construction. Also, the tourism income and tourist number both had a detectable change point at around 2006 when the Hangzhou-TIL Highway was open to traffic. Overall, the TIL region has experienced dramatic socioeconomic transformations since dam construction in the late 1950s (Fig. 8).

## Discussion

### Landscape dynamics in the TIL region after dam construction

The TIL islands and the mainland experienced a similar trend of land use transition–reforestation before 1995 from mostly sparsely-vegetated natural lands, followed by the relatively stable land uses during 1985–2005, and then tourism development for a small number of near-lakeshore islands and landscape urbanization in the surrounding mainland areas at the cost of mainly agricultural lands after 2005 (Fig. 5). In general, the landscape dynamics of the TIL-mainland system followed the conventional land use transition trajectory of deforestation-agricultural expansion-agricultural intensification-urbanization (Foley et al., 2005; Mustard et al., 2004). From 1959 when the dam was constructed to 1985, the conversion of natural lands to agricultural lands with a low level of urbanization was corroborated not only by the oral history from local people, but also in case studies of other land-bridge islands due to dam construction (Tefera & Sterk, 2008; Wijesundara & Dayawansa, 2011). Xu et al. (2016) reported that only 38.75 % of the TIL region was vegetated when the dam was constructed. Our analysis of the socioeconomic dynamics of the region (Fig. 8) also shows that the intensity of human activities, both infrastructure construction and tourism, was low before 2005, and that the reforestation prior to 1995 resulted mainly from secondary succession after the excessive deforestation during the Great Leap Forward (1958–1960) for food production. The landscape dynamics and the socioeconomical history of the region suggest that, though TIL was established in 1986 as a protected national park, the current ecological condition and landscape pattern of the TIL region are the results of both continuing natural processes and increasing human activities.

**Figure 9:**
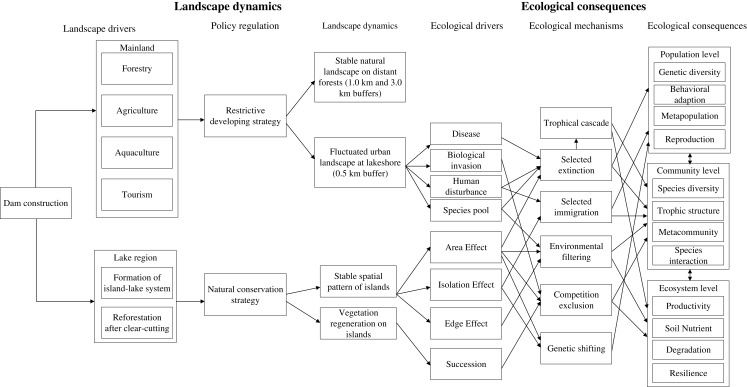
A conceptual framework that links landscape dynamics and ecological consequences since dam construction in the TIL region (modified from Fig. 5 in Wu et al., 2004). Different processes drove landscape changes on islands and the mainland which in turn affected biodiversity and ecological processes. Reforestation and natural succession led to increased greenness on the islands, whereas lakefronts were most severely affected by human activities with the shifting purposes from food production to tourism. Landscape changes near lakefronts may have affected biodiversity and ecological processes on islands via species immigration and tourist activities. Arrows indicated the potential pathways and mechanisms of how dam construction may have affected landscape dynamics and associated ecological consequences.

Under the restrictive development strategy (Fig. 9) of local government, which protected the vegetation on distant forests (1.0 and 3.0 km buffers), and developed the lakeshore zone (0.5 km buffer), human activities in the region were the most intense along the lakefront where the downtown of Chun’an County is located. The lake-island scenery created by damming provided opportunities for tourism, and local governments have invested massively on infrastructure construction to promote tourism development since 1997. In particular, to connect TIL to the Yangzi River Delta Economic Zone (the most economically developed zone in China), a highway was built in 2006, attracting many more tourists from afar. Later in 2007, a loop road around the lake was constructed, which stimulated the construction of resorts, hotels, and agritainments (farm-based tourism facilities) at the lakeshore. The fast tourism development further stimulated the local real estate industry (Yang et al., 2018). Consequently, drastic landscape urbanization and associated construction encroached into agricultural and natural lands during 2005–2010, causing more habitat fragmentation and likely decreasing habitat connectivity between islands and the surrounding mainland, and affecting the species composition on these islands. Moreover, the booming tourism has affected TIL islands ecologically (Lu & Bao, 2010). Since 2005, urban built-up areas and rural settlements have been increasing on some large TIL islands (Fig. 3), with more and more natural lands being encroached by recreational facilities, resorts, and villas.

### Ecological effects of landscape fragmentation

Landscape fragmentation by damming-induced inundation in the TIL region has resulted in myriad ecological effects on biodiversity and ecosystem processes. Most noticeably, a number of studies have reported on the negative effects of habitat loss and fragmentation on species diversity, community composition, and population and ecosystem processes. There may have been multiple mechanisms through which these fragmentation effects took place (e.g., Jones et al., 2016; Wang, Chen & Ding, 2011), and we propose that the landscape dynamics and ecological consequences in the TIL region were linked in multiple pathways and across different scales (Fig. 9).

Habitat loss due to inundation resulted in a decrease in species richness on TIL islands, which mostly have been caused by non-random extinctions of species (Si et al., 2016; Wang et al., 2010b; Wang, Chen & Ding, 2011). Other insular biogeographic studies around the world have shown that the terrestrial animals with larger size, weaker movement, and longer lifespan are more vulnerable to extinction due to dam construction (Jones et al., 2016). Environmental filtering was found to be the main mechanism for the area-related local extinction of these sensitive species as well as their functional trait distribution, genetic variation, and community similarity across islands in the TIL region (Liu et al., 2020; Liu et al., 2018; Luo et al., 2014; Si et al., 2017). The effects of fragmentation per se (without confounding area effects) indirectly influenced some basic ecological relations through selective immigration (Wang, Chen & Ding, 2011), such as the species-area relation (Hu et al., 2012) and species composition of insular biotic communities (Yu et al., 2012).

The TIL islands had lower proportions of densely-vegetated natural lands and lower NDVI values than the surrounding mainland (Figs. 5 and 8). This suggested that the post-damming secondary succession on these islands from pioneer vegetation to mature forests has been inhibited by landscape fragmentation, despite the fact that the TIL islands were less disturbed than the nearby mainland. This result is corroborated by an earlier finding that small islands were predominated by pioneer plant species (Liu et al., 2019b). Landscape fragmentation due to dam construction created mountaintop islands whose edge effects became increasingly pronounced over time (e.g., exposures to direct sunlight and wind, and frequent disturbances from waves and tides), which favored the pioneer species on the islands (Su et al., 2014). This environmental filtering, as well as the lack of the interior environment, impeded the pioneer-to-climax community transition.

### Special findings in TIL region

Most fragmentation-mediated ecological effects in the TIL region, such as area effects, isolation effects, or edge effects, on species diversity, have also been supported by studies in other dam-caused landscape worldwide (Baxter, 1977; Jones et al., 2016). However, we also find some unique and interesting results in the TIL region.

In the TIL region, we found the landscape and ecological patterns were not only dominated by ecological processes, but actually the comprehensive results of ecological-economic-social mixed effects. Different from other dam-caused landscapes, the TIL region is a very famous scenic area, hosting ~150 million tourists every year in addition to its power generation and water resource regulation responsibilities. Therefore, the long-term ecological consequences of damming is not only habitat loss and fragmentation caused by inundation, but also the shifting economic development model and life style (Wijesundara & Dayawansa, 2011). Prohibition of polluting industries and the development of local tourism regulation (National Development & Reform Commission of China, 2013) also played an important role in driving changes to the local ecosystem. Our hierarchical framework (Fig. 9) suggests that habitat fragmentation and landscape changes driven primarily by socioeconomic factors led to different ecological consequences on the TIL islands and the surrounding mainland.

Scale-dependence of the effects of habitat fragmentation is another interesting finding in TIL region. Habitat fragmentation is a landscape-scale process, but leads to impacts across multiple scales. However, few case studies have considered the fragmentation effects at multiple scales together under a hierarchical framework. In the TIL region, we found that plant traits were both affected by cross-scale interactions between the island and landscape features (Jin et al., 2020). Further, by observing interdependent effects at the plot-, island- and landscape-scales, we found that habitat fragmentation’s effects were hierarchically structured (Wilson et al., 2020). Multi-scale approaches are better than single-scale approaches to understanding the ecological impacts of damming.

We also unexpectedly found that one endangered bird (*Gorsachius magnificus*) and beetle (*Carabus davidis*) were spotted on some small and isolated islands due to the absence of predators and human activities (Li, Jing & Ding, 2007; Zhou et al., 2017), which were absent on large islands and the surrounding mainland. Finally, the role of ‘zero islands’ (island without focal taxon) has also been reconsidered when testing the small island effect in the TIL region. Specifically, excluding zero islands could lead to erroneously not detecting no small island effect (Wang et al., 2015).

Collectively these works and our study here indicate that the ecological impacts of landscape fragmentation caused by dam construction in the TIL region tend to be long-lasting. While the different mechanistic pathways proposed in our framework (Fig. 9) need to be further investigated longtermly, future research must pay greater attention to the increasing human influences the biodiversity and ecosystem processes on the islands and in the neighboring mainland landscapes. Such studies are critical for biodiversity conservation and sustainable development in the TIL region. Most insular studies have traditionally ignored the disturbance history of islands and myriad influences of the mainland (Wu et al., 2004; Wu et al., 2003b; Wu & Loucks, 1995), but our study suggests that a landscape ecological approach that integrates the lake, islands, and surrounding mainland is necessary for fully understanding the biodiversity and ecology of the TIL region. In addition, a human-environment systems perspective that focuses on landscape sustainability (Fang et al., 2018; Wu, 2013; Zhou, Wu & Anderies, 2019) is required to make such studies directly relevant to policy-making and regional planning.

## Conclusions

Damming is one of the most destructive human disturbances to nature, profoundly impacting biodiversity and ecosystem processes around the world (Li et al., 2013; Schmutz & Moog, 2018). Though they do not promote damming, ecologists have taken advantage of a number of artificial archipelagos formed by damming to study the ecological impacts of habitat/landscape fragmentation which is a primary concern of our time. The Thousand Island Lake is a quintessential example. After more than 60 years since the dam construction, the ecological impacts of damming and landscape fragmentation have been numerous and profound, but so far studies have been sporadic and focused only on a small set of islands. Also, most existing studies took the traditional island biogeographic approach, without adequate consideration of within-island spatial heterogeneity, edge effects, island-mainland landscape dynamics and the impacts from socioeconomic development. To address these problems and promote TIL as a “natural experiment” for answering important questions on landscape fragmentation (Wilson et al., 2016), this study provides the first quantitative analysis of landscape patterns of both islands and the surrounding mainland. We also have shown mounting evidence of myriad landscape fragmentation effects on the biodiversity and ecosystem processes in the region, and pointed out future research directions. We suggest that future ecological research in the TIL region should emphasize human influences and consider the islands and neighboring mainland together as an integrated human-environment system. Such an approach is necessary not only for better understanding fragmentation ecology, but also for promoting the environmental sustainability of archipelagos that are intrinsically connected to their neighboring mainland.

## Supplemental Information

10.7717/peerj.11416/supp-1 Supplemental Information 1 Schema for reclassifying land use/cover classes in Liu et al. (2005).

10.7717/peerj.11416/supp-2 Supplemental Information 2 List of relevant ecological publications on the Thousand Island Lake.

10.7717/peerj.11416/supp-3 Supplemental Information 3 Computer code for NDVI calculation from GEE.

## Additional Information and Declarations

### Data Availability

The following information was supplied regarding data availability: The raw data and codes generated or analyzed during this study are available in the article and Supplemental Files. The LUC data is available at the Resources and Environment Sciences Data Center (http://www.resdc.cn) and can be accessed by contacting customer service (xuxl@lreis.ac.cn) or by registering at the website.
