# Supplementary material for: Spatiotemporal patterns and ecological consequences of a fragmented landscape created by damming
Source: PeerJ. 2021 May 21;9:e11416. doi: 10.7717/peerj.11416 (PMC8142928; doi:10.7717/peerj.11416)
Supplement: Supplemental Information 1 [file peerj-09-11416-s001.docx]

**Appendix 1. Schema for reclassifying land use/cover classes in Liu et al. (2005).**

| Reclassified Category | Original classification | Land use/cover description from Liu et al. 2005 |
| --- | --- | --- |
| Agricultural lands | Paddy land | Cropland that has enough water supply and irrigation facilities for planting paddy rice, lotus etc., including rotation land for paddy rice and dry farming crops. |
|  | Dry land | Cropland for cultivation without water supply and irrigating facilities; cropland that has water supply and irrigation facilities and planting dry farming crops; cropland planting vegetables; fallow land. |
| Industrial and transportation lands | Built-up land (others) | Lands used for factories, quarries, mining, oil-field slattern outside cities and lands for special uses such as transportation and airport. |
| Rural settlement | Rural settlements | Lands used for settlements in villages. |
| Urban built-up lands | Urban built-up | Lands used for urban. |
| Sparsely-vegetated natural lands | Woods | Lands covered by trees with canopy cover between 10– 30%. |
|  | Woodland (others) | Lands such as tea-garden, orchards, groves and nurseries. |
|  | Moderate grass | Grassland with canopy coverage between 20% and 50%. |
|  | Sparse grass | Grassland with canopy cover between 5% and 20%. |
|  | Permanent ice and snow | Lands covered by perennial snowfields and glaciers. |
|  | Sandy land | Sandy land covered with less than 5% vegetation cover. |
|  | Gobi | Gravel covered land with less than 5% vegetation cover. |
|  | Salina | Lands with salina accumulation and sparse vegetation. |
|  | Bare soil | Bare exposed soil with less than 5% vegetation cover. |
|  | Bare rock | Bare exposed rock with less than 5% vegetation cover. |
|  | Unused land (others) | Other lands such as alpine desert and tundra. |
| Densely-vegetated natural lands | Forest | Natural or planted forests with canopy cover greater than 30%. |
|  | Shrub | Lands covered by trees less than 2 m high, the canopy cover >40%. |
|  | Dense grass | Grassland with canopy coverage greater than 50%. |
| Water bodies | Stream and rivers | Lands covered by rivers including canals. |
|  | Lakes | Lands covered by lakes. |
|  | Reservoir and ponds | Man-made facilities for water reservation. |
|  | Beach and shore | Lands between high tide level and low tide level. |
|  | Bottomland | Lands between normal water level and flood level. |
|  | Swampland | Lands with a permanent mixture of water and herbaceous or woody vegetation that cover extensive areas. |
