# Supplementary material for: Spatiotemporal patterns and ecological consequences of a fragmented landscape created by damming
Source: PeerJ. 2021 May 21;9:e11416. doi: 10.7717/peerj.11416 (PMC8142928; doi:10.7717/peerj.11416)
Supplement: Supplemental Information 2 [file peerj-09-11416-s002.docx]

**Supporting Information for Hu et al.: Spatiotemporal patterns and ecological consequences of a fragmented landscape created by damming**

**Appendix 2. List of relevant ecological publications on the Thousand Island Lake.**

1. Chen CW, Holyoak M, Si XF, Wang YP, and Ding P. 2018. Do seasonal species assemblages differ in their biogeography? Evidence from the spatial structure of bird communities on land-bridge islands. *Journal of Biogeography* 45: 473-483.
2. Ding L, Lu J, Xu G, and Wu J. 2004. Effects of ecological protection and development on landscape pattern in the Thousand-Island Lake region, Zhejiang Province. *Biodiversity Science*: 473-480.
3. Ding L, Lu J, Zhao Y, and Liu B. 2006. Edge effects of landscape fragmentation on the shrub-layer vegetation in the One-Thousand Island Lake. *Journal of Zhejiang University (Agriculture and Life Sciences)*: 563-568.
4. Ding ZF, Feeley KJ, Hu HJ, and Ding P. 2015. Bird guild loss and its determinants on subtropical land-bridge islands, China. Avian Research 6: 1-9.
5. Ding ZF, Feeley KJ, Wang YP, Pakeman RJ, and Ding P. 2013. Patterns of bird functional diversity on land-bridge island fragments. *Journal of Animal Ecology* 82: 781-790.
6. Hu G, Feeley KJ, Wu JG, Xu GF, and Yu MJ. 2011. Determinants of plant species richness and patterns of nestedness in fragmented landscapes: evidence from land-bridge islands. *Landscape Ecology* 26: 1405-1417.
7. Hu G, Feeley KJ, and Yu MJ. 2016. Habitat Fragmentation Drives Plant Community Assembly Processes across Life Stages. *Plos One* 11: e0159572.
8. Hu G, Wu JG, Feeley KJ, Xu GF, and Yu MJ. 2012. The Effects of Landscape Variables on the Species-Area Relationship during Late-Stage Habitat Fragmentation. *Plos One* 7(8): e43894.
9. Hu G., Wilson M.C., Wu J., Yu J. and Yu M. 2019. Decoupling species richness variation and spatial turnover in beta diversity across a fragmented landscape. PeerJ 7: e6714.
10. Huang J, Hu G, Yuan J, and Luo Y. 2013. A comparison of pitfall trapping and the Winkler method for investigating soil arthropod diversity: a case study on the closed habitats of land-bridge islands. *Chinese Journal of Applied Entomology* 50: 1679-1691.
11. Jia XQ, He ZH, Weiser MD, Yin T, Akbar S, Kong XS, Tian K, Jia YY, Lin H, Yu MJ, and Tian XJ. 2016. Indoor evidence for the contribution of soil microbes and corresponding environments to the decomposition of *Pinus massoniana* and *Castanopsis sclerophylla* litter from Thousand Island Lake. *European Journal of Soil Biology* 77: 44-52.
12. Jin Y, Didham RK, Yuan J, Hu G, Yu J, Zheng S, and Yu M. 2020. Cross-scale drivers of plant trait distributions in a fragmented forest landscape. *Ecography* 43: 467-479.
13. Li BC, Jing PP, and Ding P. 2007. First breeding observations and a new locality record of white-eared night-heron Gorsachius magnificus in southeast China. *Waterbirds* 30: 301-304.
14. Li T, and Xu Z. 2017. A new species of Loboscelidia (Hymenoptera: Chrysididae: Loboscelidiinae) from China. *Entomotaxonomia* 39: 163-168.
15. Liu C, Ding Z, and Ding P. 2015. Seasonal changes in sensitivity of bird guilds to habitat fragmentation on landbridge islands in the Thousand Island Lake, China. *Acta Ecologica Sinica* 35: 6759-6768.
16. Liu J, Bao Y, Zhang X, Lin J, Ye B, and Wang Y. 2012. Population genetics of Niviventer confucianus and its relationships with habitat area in Thousand Island Lake region. *Acta Ecologica Sinica* 32: 758-766.
17. Liu J, Bao Y, Wang Y, Sun B, and Ye B. 2013. Effects of islanding on the genetics of Niviventer confucianus (Mamalia: Rodentia: Muridae) populations in the Thousand Island Lake region. *Journal of Natural History* 47: 2583-2598.
18. Liu J, Coomes DA, Hu G, Liu J, Yu J, Luo Y, and Yu M. 2019. Larger fragments have more late-successional species of woody plants than smaller fragments after 50 years of secondary succession. *Journal of Ecology* 107: 582-594.
19. Liu J, Matthews TJ, Zhong L, Liu J, Wu D, and Yu M. 2020. Environmental filtering underpins the island species-area relationship in a subtropical anthropogenic archipelago. *Journal of Ecology* 108: 424-432.
20. Liu J, Slik F, Coomes DA, Corlett RT, Wang Y, Wilson MC, Hu G, Ding P, and Yu. 2019b2019c. The distribution of plants and seed dispersers in response to habitat fragmentation in an artificial island archipelago. *Journal of Biogeography* 46:1152-1162.
21. Liu J, Vellend M, Wang ZH, and Yu M. 2018. High beta diversity among small islands is due to environmental heterogeneity rather than ecological drift. *Journal of Biogeography* 45: 2252-2261.
22. Lu J, Ding L, and Xu G. 2005. Effects of islanding on plant species diversity in Thousand-island Lake region. *Chinese Journal of Applied Ecology*: 1672-1676.
23. Lu JB, Jiang L, Yu L, and Sun Q. 2011. Local Factors Determine Plant Community Structure on Closely Neighbored Islands. *Plos One* 6(5): e19762.
24. Luo Y, Liu J, Huang J, and Bai M. 2014. Genetic diversity of Lycosa coelestris on islands in the Thousand Island Lake (TIL) and the effects of habitat fragmentation*. Chinese Science Bulletin* 59: 1851-1860.
25. Luo Y, Liu J, Huang J, and Bao H. 2013. Effects of habitat fragmentation on the genetic diversity of Pachycondyla luteipes on islands in the Thousand Island Lake, East China. *Acta Ecologica Sinica* 33: 6041-6048.
26. Luo Y, Yu M, Yu J, Zheng S, Liu J, and Yu M. 2017. Effects of plant traits and the relative abundance of common woody species on seedling herbivory in the Thousand Island Lake region. *Chinese Journal of Plant Ecology* 41: 1033-1040.
27. Lv K, Zhou J, Gu JQ, Zhou GX, Wang W, and Xu ZH. 2018. Habitat fragmentation influences gene structure and gene differentiation among the *Loxoblemmus aomoriensis* populations in the Thousand Island Lake. *Mitochondrial DNA Part A* 29: 222-227.
28. Nan G, Jin Y, Wu C, Xu G, Cao N, Li M, and Yu M. 2018. Effects of habitat fragmentation on the soil seed banks of Masson pine (*Pinus massoniana*) forests in the Thousand Island Lake region，eastern China. *Acta Ecologica Sinica* 38: 206-214.
29. Peng S, Hu G, and Yu M. 2014. Beta diversity of vascular plants and its influencing factors on islands in the Thousand Island Lake. *Acta Ecologica Sinica* 34: 3866-3872.
30. Ren L, Xu Z, Lu J, Zhao G, and Zhang Q. 2009. Relationships between island characteristics and arthropod diversity in Thousand-island Lake. *Chinese Journal of Applied Ecology* 20: 2255-2261.
31. Rong F, Wang Z, Wu C, Xu G, Xu J, Li M, and Yu M. 2018. Effects of habitat fragmentation on soil properties of the secondary Masson pine (*Pinus massoniana*) forests of the Thousand Island Lake. *Journal of Zhejiang University (Science Edition)* 45: 748-755+764.
32. Shen L, Bao Y, Zhang X, Wei D, and Liu J. 2011. Effect of different seasons and sex of *Niviventer confucianus* on islands at Thousand Island Lake. *Journal of Zhejiang Normal University (Natural Sciences)* 34: 328-332.
33. Si XF, Baselga A, Leprieur F, Song X, and Ding P. 2016. Selective extinction drives taxonomic and functional alpha and beta diversities in island bird assemblages. *Journal of Animal Ecology* 85: 409-418.
34. Si XF, Cadotte MW, Zeng D, Baselga A, Zhao YH, Li JQ, Wu YR, Wang SY, and Ding P. 2017. Functional and phylogenetic structure of island bird communities. *Journal of Animal Ecology* 86: 532-542.
35. Si XF, Cadotte MW, Zhao YH, Zhou HN, Zeng D, Li JQ, Jin TH, Ren P, Wang YP, Ding P, and Tingley MW. 2018. The importance of accounting for imperfect detection when estimating functional and phylogenetic community structure. *Ecology* 99: 2103-2112.
36. Si XF, Pimm SL, Russell GJ, and Ding P. 2014. Turnover of breeding bird communities on islands in an inundated lake. *Journal of Biogeography* 41: 2283-2292.
37. Song X, Holt RD, Si XF, Christman MC, and Ding P. 2018. When the species-time-area relationship meets island biogeography: Diversity patterns of avian communities over time and space in a subtropical archipelago. *Journal of Biogeography* 45: 664-675.
38. Su X, Yuan J, Hu G, Xu G, and Yu M. 2014. Edge effect of the plant community structure on land-bridge islands in the Thousand Island Lake. *Chinese Journal of Applied Ecology* 25: 77-84.
39. Sun B, Bao Y, Zhang L, and Zhao Q. 2009. Age-structure and reproduction investigation on Niviventer confucianus living on islands at Qiandao Lake in autumn. *Acta Theriologica Sinica* 29: 269-276.
40. Sun B, Bao Y, Zhang L, Zhao Q, and Hu Z. 2009. Preliminary Study on Relative Fatness of Niviventer confucianus on Islands of Qiandao Lake Region. *Zoological Research* 30: 545-552.
41. Sun J, Wang S, Wang Y, Shao D, and Ding P. 2011. Effects of habitat fragmentation on avian nest predation risk in Thousand Island Lake, Zhejiang Province. *Biodiversity Science* 19: 528-534.
42. Sun Q, Lu J, Wu J, and Zhang F. 2008. Effects of island area on plant species distribution and conservation implications in the Thousand Island Lake region. *Biodiversity Science*: 1-7.
43. Tan S, Hu G, Shao D, Hu R, Xu G, and Yu M. 2010. Floristic analysis of seed plants in the Thousand Island Lake region. *Guihaia* 30: 770-775+752.
44. Tian Y, Jin Y, Wang Z, Su X, Hu G, Xu L, and Yu M. 2016. Seedling dynamics of shade tolerant and intolerant woody plants in the Masson pine forests on islands of the Thousand Island Lake. *Journal of Zhejiang University (Science Edition)* 43: 426-435.
45. Tong X, Zhang YX, Wang R, Inbar M, and Chen XY. 2017. Habitat fragmentation alters predator satiation of acorns. *Journal of Plant Ecology* 10: 67-73.
46. Wang X, Wang Y, and Ding P. 2012. Nested species subsets of amphibians and reptiles in Thousand Island Lake. *Zoological Research* 33: 439-446.
47. Wang Y, Zhang J, Feeley KJ, Jiang P, and Ding P. 2009. Life-history traits associated with fragmentation vulnerability of lizards in the Thousand Island Lake, China. *Animal Conservation* 12: 329-337.
48. Wang YP, Bao YX, Yu MJ, Xu GF, and Ding P. 2010. Nestedness for different reasons: the distributions of birds, lizards and small mammals on islands of an inundated lake. *Diversity and Distributions* 16: 862-873.
49. Wang YP, Chen SH, and Ding P. 2011. Testing multiple assembly rule models in avian communities on islands of an inundated lake, Zhejiang Province, China. *Journal of Biogeography* 38: 1330-1344.
50. Wang YP, Thornton DH, Ge DP, Wang SY, and Ding P. 2015. Ecological correlates of vulnerability to fragmentation in forest birds on inundated subtropical land-bridge islands. *Biological Conservation* 191: 251-257.
51. Wang YP, Wang X, and Ding P. 2012. Nestedness of snake assemblages on islands of an inundated lake. *Current Zoology* 58: 828-836.
52. Wang YP, Wang X, Wu Q, Chen CS, Xu AC, and Ding P. 2018. The small-island effect in amphibian assemblages on subtropical land-bridge islands of an inundated lake. *Current Zoology* 64: 303-309.
53. Wang YP, Wu Q, Wang X, Liu C, Wu LB, Chen CW, Ge DP, Song X, Chen CS, Xu AC, and Ding P. 2015. Small-island effect in snake communities on islands of an inundated lake: The need to include zeroes. *Basic and Applied Ecology* 16: 19-27.
54. Wang YP, Zhang M, Wang SY, Ding ZF, Zhang JC, Sun JJ, Li P, and Ding P. 2012. No evidence for the small-island effect in avian communities on islands of an inundated lake. *Oikos* 121: 1945-1952.
55. Wang YY, Zhu YQ, and Wang YF. 2012. Differences in spatial genetic structure and diversity in two mosses with different dispersal strategies in a fragmented landscape. *Journal of Bryology* 34: 9-16.
56. Wilson MC, Chen XY, Corlett RT, Didham RK, Ding P, Holt RD, Holyoak M, Hu G, Hughes AC, Jiang L, Laurance WF, Liu JJ, Pimm SL, Robinson SK, Russo SE, Si XF, Wilcove DS, Wu JG, and Yu MJ. 2016. Habitat fragmentation and biodiversity conservation: key findings and future challenges. *Landscape Ecology* 31: 219-227.
57. Wilson MC, Hu G, Jiang L, Liu J, Liu J, Jin Y, Yu M, and Wu J. 2020. Assessing habitat fragmentation's hierarchical effects on species diversity at multiple scales: the case of Thousand Island Lake, China. *Landscape Ecology* 35:529-544.
58. Wu LB, Si XF, Didham RK, Ge DP, and Ding P. 2017. Dispersal modality determines the relative partitioning of beta diversity in spider assemblages on subtropical land-bridge islands. *Journal of Biogeography* 44: 2121-2131.
59. Wu Q, Wang Y, and Ding P. 2015. Ontogenetic Shifts in Diet of the Piebald Odorous Frog Odorrana schmackeri in the Thousand Island Lake. *Chinese Journal of Zoology* 50: 204-213.
60. Wu Y, Si X, Chen C, Zen D, Zhao Y, Li J, and Ding P. 2016. Effects of dispersal abilities on community dynamics of breeding birds on the land-bridge islands in the Thousand Island Lake, China. *Biodiversity Science* 24: 1135-1145.
61. Xu A, Si X, Wang Y, and Ding P. 2014. Camera traps and the minimum trapping effort for ground-dwelling mammals in fragmented habitats in the Thousand Island Lake, Zhejiang Province *Biodiversity Science* 22: 764-772.
62. Xu G, Hu G, Si X, Lu G, Yu M, Ding P, and Hong L. 2015. Habitat Fragmentation and Biodiversity Ｒesearch on Thousand Island Lake. *Forest Inventory and Planning* 40: 42-48.
63. Ye B, Bao Y, Wang Y, Zhang S, and Fang P. 2016. Genetic structure and relatedness based on parentage analysis in family groups of Chinese white-bellied rats (*Niviventer confucianus*) in Thousand Island Lake. *Acta Ecologica Sinica* 36: 811-820.
64. Ye B, Shen L, Bao Y, and Zhang X. 2013. Effects of age，sex and season on the maximum movement distance of *Niviventer confucianus* in Thousand Island Lake. *Acta Ecologica Sinica* 33: 3311-3317.
65. Yu J, Jin Y, Zheng S, Hu G, Liu J, Yuan J, Liu J, and Yu M. 2017. Differentiation in leaf and branch traits of angiosperms and their relationships between species abundance in the Thousand Island Lake Region. *Journal of Zhejiang University (Science Edition)* 44: 437-445.
66. Yu L, and Lu JB. 2011. Does Landscape Fragmentation Influence Sex Ratio of Dioecious Plants? A Case Study of *Pistacia chinensis* in the Thousand-Island Lake Region of China. *Plos One* 6(8): e22903.
67. Yu MJ, Hu G, Feeley KJ, Wu JG, and Ding P. 2012. Richness and composition of plants and birds on land-bridge islands: effects of island attributes and differential responses of species groups. *Journal of Biogeography* 39: 1124-1133.
68. Yuan N, Comes HP, Cao YN, Guo R, Zhang YH, and Qiu YX. 2015. A comparative study on genetic effects of artificial and natural habitat fragmentation on *Loropetalum chinense* (Hamamelidaceae) in Southeast China. *Heredity* 114: 544-551.
69. Yuan N, Comes HP, Mao YR, Qi XS, and Qiu YX. 2012. Genetic effects of recent habitat fragmentation in the thousand-island lake region of southeast china on the distylous herb *Hedyotis chrysotricha* (Rubiaceae). *American Journal of Botany* 99: 1715-1725.
70. Yuan N, Sun Y, Nakamura K, and Qiu YX. 2012. Development of microsatellite markers in heterostylous *Hedyotis chrysotricha* (Rubiaceae). *American Journal of Botany* 99: E43-E45.
71. Zhang J, Wang Y, Jiang P, Li P, Yu M, and Ding P. 2008. Nested analysis of passeriform bird assemblages in the Thousand Island Lake region. *Biodiversity Science*: 321-331.
72. Zhang M, Sun J, Wang Y, Jiang P, Ding P, and Xu G. 2010. Effects of habitat fragmentation on the use of nest site resources by great tits in Thousand Island Lake, Zhejiang Province. *Biodiversity Science* 18: 383-389.
73. Zhang X, Bao Y, Liu J, Shen L, Ye B, and Shi W. 2013. The research on the age structure and sex ratio of *Niviventer confucianus* in Thousand Island Lake. *Acta Ecologica Sinica* 33: 5000-5007.
74. Zhang X, Bao Y, Liu J, Shen L, Zhang S, and Fang P. 2013. Population dynamics of *Niviventer confucianus* in Thousand Island Lake. *Acta Ecologica Sinica* 33: 4665-4673.
75. Zhang X, Wang H, Bao Y, Wang Y, and Ye B. 2016. Dispersal characteristics of populations of Chinese white-bellied rats (*Niviventer confucianus*) in the Thousand Island Lake region. *Acta Ecologica Sinica* 36: 1312-1320.
76. Zhou W, Luo Y, Zhong L, and Pan L. 2017. Effects of habitat fragmentation on species diversity of ground dwelling beetles in the Thousand-Island Lake region, Zhejiang, China. *Chinese Journal of Applied Ecology* 28: 509-518.
77. Zhou W, Zhong L, Huang J, Xie Y, and Luo Y. 2019. Species diversity, functional group structure, and influencing factors of spider community in fragmented landscape of Thousand Island Lake. *Acta Ecologica Sinica*: 1-11.
78. Zhu L, Lu J, and Yu L. 2010. Effects of island area and distances among islands on their β-diversity of plant species in One-thousand Island Lake region. *Journal of Zhejiang University (Agriculture and Life Sciences)* 36: 691-698.
