# Supplementary material for: Spatiotemporal patterns and ecological consequences of a fragmented landscape created by damming
Source: PeerJ. 2021 May 21;9:e11416. doi: 10.7717/peerj.11416 (PMC8142928; doi:10.7717/peerj.11416)
Supplement: Supplemental Information 3 [file peerj-09-11416-s003.docx]

**Supporting Information for Hu et al.: Spatiotemporal patterns and ecological consequences of a fragmented landscape created by damming**

**Appendix 3. Computer code for NDVI calculation from GEE**

//---------------------------------import chun’an shapefile------------------------------------------------

Imports (1 entry)

var chun_an: FeatureCollection (1 element)

type: FeatureCollection

columns: Object (1 property)

features: List (1 element)

//--------------------To calculate NDVI from Landsat 5 data-----------------------------------------

//---------------------------------MaskCloud--------------------------------------------------------

//function: mask cloud

//This demonstrates the use of the pixel QA band to mask

// clouds in surface reflectance (SR) data. It is suitable

// for use with any of the Landsat SR datasets.

// Function to cloud mask from the pixel_qa band of Landsat 5 SR data.

function maskL5sr(image)

{

var timeStart = image.get('system:time_start');

var srImageList = ee.ImageCollection('LANDSAT/LT05/C01/T1_SR')

.filterMetadata('system:time_start','equals',timeStart)

.toList(5);

var qa = image.select('pixel_qa');

// If the cloud bit (5) is set and the cloud confidence (7) is high

// or the cloud shadow bit is set (3), then it's a bad pixel.

var cloud = qa.bitwiseAnd(1 << 5)

.and(qa.bitwiseAnd(1 << 7))

.or(qa.bitwiseAnd(1 << 3))

// Remove edge pixels that don't occur in all bands

var mask = image.mask().reduce(ee.Reducer.min());

return image.updateMask(cloud.not()).updateMask(mask);

}

//----------------------------Calculate&AddBands---------------------------------------------

// calculate FAI&lswi - note the bands are different for LC8

// multiply 0.0001 is needed because the SR data is scaled by 10000

function ND_VI(image,b1,b2,bName)

{

var VI = image.normalizedDifference([b1,b2]).rename(bName);

return VI.updateMask(VI.gt(-1).and(VI.lt(1)));

}

function addLandsatVIs(img)

{

var NDVI = ND_VI(img,'B4','B3','NDVI');

return img.addBands(NDVI);

}

/////////////////////////////////////calculate VIs///////////////////////////

for (var yr= 1985; yr <= 2018; yr = yr + 1) {

print('This is year' + yr)

print(yr)

var start_date = ee.Date.fromYMD(yr,1,1);

var end_date = ee.Date.fromYMD(yr,12,31);

var studyArea = ee.FeatureCollection(chun_an);

// Map.addLayer(studyArea);

//---------------------------------------------cal VIs---------------------------------------------------------------

//get Landsat5 collction

var collection_L5 = ee.ImageCollection('LANDSAT/LT05/C01/T1_SR')

.filterBounds(studyArea)

.filterDate(start_date, end_date)

.map(maskL5sr)

.select(

['B1', 'B2', 'B3', 'B4', 'B5', 'B7', 'pixel_qa'])

.map(addLandsatVIs);

print('Landsat5')

print(collection_L5.size());

//cal maximum ndvi

var max_ndvi_L5 = collection_L5.select('NDVI').reduce(ee.Reducer.max())

Map.addLayer(max_ndvi_L5.clip(studyArea),{}, 'max_ndvi_L5_'+yr,true);

//export calculated NDVI

Export.image.toDrive({

image: max_ndvi_L5.clip(studyArea),

description: 'Max_NDVI_L5_'+yr,

scale: 30,

folder:'L5_NDVI',

maxPixels: 2e12

});

}
